# Epidemiology of *Staphylococcus haemolyticus* nosocomial bacteraemia in neonatal intensive care units, France, 2019 to 2023: predominance of the ST29 (CC3) multidrug-resistant lineage

**DOI:** 10.2807/1560-7917.ES.2025.30.11.2400309

**Published:** 2025-03-20

**Authors:** Patricia Martins Simões, Nathalie van der Mee-Marquet, Benjamin Youenou, Anne-Gaelle Ranc, Céline Dupieux-Chabert, Guillaume Menard, Clarisse Dupin, Marine Butin, François Vandenesch, Frédéric Laurent, Anne Berger-Carbonne, Camille Kolenda, Anne Tristan, S. haemolyticus neonatal infections Study Group, S. haemolyticus neonatal infections Study Group, Pascale Bailly, Christine Barreto, Clémence Beauruelle, Carole Benet, Elise Bernard, Xavier Bertrand, Pascal Boileau, Nadège Bourgeois-Nicolaos, Sandrine Canouet, Laurent Cavalie, Camille Chagneau, Aminata Cisse, Mireille Cheron, Véronique Deroin, Pierre-Yves Donnio, Damien Dubois, Yann Dumont, Pascal Fascia, Tiphanie Faïs, Agnès Gaudichon, Laure Gibert, Florence Grattard, Béatrice Grisi, Nadia Idri, Franck Labbé, Emilie Lafeuille, Claudie Lamoureux, Etienne Laurens, Christine Lawrence, Stéphanie Lefflot, Hervé Le Bars, Stéphane Le Bouedec, Emmanuel Lecorche, Sylvie Ledru, Elisabeth Le Glass, Philippe Lehours, Carole Lemarié, Hervé Lecuyer, David Leyssene, Mathilde Liberge, Eugénie Maurin, Céline Ménard, Isabelle Mézard, Chantal Miquel, Emmanuelle Motte-Signoret, Cécile Mourlan, Anaëlle Muggeo, Hélène Pailhoriès, Céline Plainvert, Sara Romano-Bertrand, Marlène Sauget, Florence Stordeur, Asmaa Tazi, Sylvie Vacher, Paul Verhoeven, Véronique Vernet-Garnier

**Affiliations:** 1 Centre National de Référence des Staphylocoques, Institut des agents infectieux, Hospices Civils de Lyon, Lyon, France; 2 Centre International de Recherche en Infectiologie (CIRI), Université de Lyon, Inserm U1111, Université Claude Bernard Lyon 1, CNRS UMR5308, ENS de Lyon, Lyon, France; 3 National Network for Surveillance and Prevention of Infections Associated with Invasive Devices (SPIADI Network), Centre d'Appui Pour la Prévention des Infections Associées Aux Soins (Cpias) Centre Val de Loire, Hôpital Bretonneau, Centre Hospitalier Régional Universitaire, Tours, France; 4 CHU de Rennes, service de bactériologie-hygiène hospitalière, 35033 Rennes, France; 5 Université de Rennes, UMR_S 1230 INSERM BRM, Rennes, France; 6 Service de Microbiologie, CH de St BRIEUC, Saint-Brieuc, France; 7 Service de Réanimation Néonatale, HFME, Hospices Civils de Lyon, Bron, France; 8 Direction des maladies infectieuses, Santé Publique France, Saint-Maurice, France; 9 The members of the Study Group are listed under Collaborators.

**Keywords:** *Staphylococcus haemolyticus*, neonatal infections, bacteraemia, epidemiology, multi-drug resistance, neonatal intensive care units, outbreak, bloodstream infections

## Abstract

**Background:**

*Staphylococcus haemolyticus* (SH) is an opportunistic pathogen associated with nosocomial infections, particularly bacteraemia in neonates. Epidemiological trends and genetic diversity of these infections worldwide are largely unknown.

**Aim:**

To investigate an increase in SH vascular catheter-related bacteraemia in neonates and describe the molecular epidemiology in France between 2019 and 2023.

**Methods:**

We analysed clinical and microbiological surveillance data from the French national surveillance network for central catheter-related (venous and umbilical) infections between 2019 and 2023. We also performed genomic and phylogenetic analyses of 496 strains isolated both inside (n = 383 from neonates, staff and environmental samples) and outside (n = 113 from adults) the neonatal intensive care unit (NICU) settings.

**Results:**

The proportion of SH among the 474 reported cases of nosocomial bacteraemia increased from about 20% to 30% over 5 years, mainly affecting very low birth weight preterm neonates (≤ 1,500 g). The ST29 sequence type (ST) not prevalent in previous studies was predominant, accounting for 74% of NICU strains. ST29 was characterised by phenotypic multidrug resistance to at least six classes of antibiotics (oxacillin, quinolones, gentamicin, cotrimoxazole, clindamycin and rifampicin), which distinguished it with good sensitivity and specificity from other prevalent multidrug-resistant STs identified (ST1 and ST25). ST29 strains more frequently harboured the *drfG*, *vga-LC* and *mupA* genes and a triple point mutation (D471E, I527M and S532N) in the *rpoB* gene.

**Conclusions:**

The present study highlights the success of a highly resistant ST29 lineage in French NICUs mainly affecting very low birth weight premature neonates.

**Key public health message**

**What did you want to address in this study and why?**

Life expectancy of very low birth weight (VLBW) preterm neonates has increased because of improvements in both technology and care. Nevertheless, bacterial infections remain a leading cause of complications and death, and are frequently caused by multidrug-resistant *Staphylococcus haemolyticus* (SH). We characterised SH infections in preterm neonates in France from 2019 to 2023 using national surveillance data of hospital-acquired bacteraemia.

**What have we learnt from this study?**

The number of VLBW neonates increased in the 5-year period, with the proportion of SH infections increasing from 20% to 30% among over 450 cases of bacteraemia. Genomic analysis showed a predominance of SH lineage ST29. This lineage was characterised by a particular antimicrobial resistance pattern distinct from other lineages and could be used as a screening marker for infection control interventions.

**What are the implications of your findings for public health?**

French learned societies for microbiology, neonatal medicine and infection prevention were informed by these results, leading to the publication of new recommendations to improve the diagnosis, treatment and prevention of *S. haemolyticus* bacteraemia in neonatal intensive care units. The surveillance of these infections should be extended to other European countries.

## Introduction

In recent years, skin commensal *Staphylococcus haemolyticus* (SH) has gained increasing attention as an emerging multidrug-resistant (MDR) opportunistic pathogen and as one of the most frequently isolated coagulase-negative staphylococci (CoNS) species associated with nosocomial infections alongside *S. epidermidis* [1]. It is notably associated with nosocomial bacteraemia in very low birth weight preterm neonates, who require long-term hospitalisation in neonatal intensive care units (NICUs) and the continuous usage of indwelling medical devices, such as central lines/catheters and other intravascular devices [2,3]. 

The epidemiological trends and genetic diversity of SH infections worldwide remain largely unknown. Previously described nosocomial SH strains belong to the highly prevalent and genetically diverse clonal complex CC3 [4], which includes sequence types ST1, ST3 and ST42, identified in both NICU and non-NICU settings [5-8]. Frequent recombination events, caused by the high number of insertion sequences (IS) and the variability in the *OriC* environment [5-7,9], and horizontal gene transfer are the main drivers of SH genomic plasticity. Their pathogenicity is probably related to the presence of certain enzymes, cytolysins such as phenol soluble modulins (PSMs), adhesins and their ability to form thick biofilms [10,11]. Because SH can form biofilms [11], persist and thrive in the hospital environment [12], and easily acquire antibiotic resistance [13], MDR strains have emerged and given rise to nosocomial endemic clades in the last decade [8,9,14], notably in NICUs [9,14]. 

In late 2021 and early 2022, several French hospitals with NICUs reported grouped cases of SH nosocomial infections to the French health authorities (SpF), which issued a national alert for all hospitals with NICUs and requested the expertise of the French National Reference Centre for Staphylococci (FNRCS) to investigate the cases. In this study, we utilised data from the French national network for the monitoring and prevention of infections associated with invasive devices (*Surveillance et Prévention des Infections Associées aux Dispositifs Invasifs*, SPIADI), with the aim of examining the proportion of SH catheter-related bacteraemia in neonates in France between 2019 and 2023 in relation to clinical data. In addition, we investigated the molecular epidemiology of SH isolates sent to the FNRCS during the same period in order to understand the molecular diversity and characterise potential SH clones associated with bacteraemia in premature neonates.

## Methods

### Surveillance of catheter-related bacteraemia in France

Since 2019, the French Ministry of Health has mandated that all hospitals in France require their local infection prevention teams to implement the 2022**–**2025 National Strategy for Infection Prevention and Antibiotic Resistance. Under the auspices of SpF, the SPIADI network oversees the surveillance of catheter-related infections at the national level**,** following a methodology derived from the European Centre for Disease Prevention and Control (ECDC) Healthcare-Associated Infections surveillance Network (HAI-Net) ICU protocol (v2.2) [15]. Each year, the SPIADI team invites all French intensive care, surgical, medical, obstetric, emergency, rehabilitation, dialysis and long-term care units to participate in a 3-month surveillance of bacteraemia between 1 January and 15 July. 

In the present study, we used data from the SPIADI surveys between 2019 and 2023 on catheter-related bacteraemia reported exclusively from NICUs. Non-nosocomial bacteraemia cases were excluded from the SPIADI surveillance.

### Case diagnosis and study enrolment

The diagnosis of nosocomial bacteraemia was based on the analysis of any positive blood culture by a local infection control practitioner [15]. Bacteraemia was confirmed if the isolated microorganism was not a potential contaminant. In case of isolation of a skin commensal species, at least two positive cultures of the same species taken at different times during the same episode were necessary to rule out contaminations. Alternatively, if only one positive culture was available, additional criteria were considered including improvement following the initiation of targeted antibiotic treatment or catheter removal, as well as clinical and/or biological signs of infection, i.e. fever, elevated C-reactive protein (CRP). 

Neonates were enrolled in the study through either a unit- or a patient-based survey, depending on the unit’s choice [15]. In the unit-based survey, characteristics of the neonates diagnosed with bacteraemia (sex, birth weight, gestational age in weeks of amenorrhoea, use of central venous catheters and umbilical catheters, death within 7 days of diagnosis) and of the corresponding bacteraemia episodes (suspected origin i.e. skin, surgical site, lungs, urinary tract, intravascular device, intra-abdominal or digestive tract, the bacterial species, time between entry into the NICU and onset of infection) were collected. In the patient-based survey, the collection of data (sex, birth weight, gestational age in weeks of amenorrhoea, use of central lines) was conducted for all neonates hospitalised during the 3-month survey period, whether they developed nosocomial bacteraemia or not.

### *Staphylococcus haemolyticus* isolates and phenotypic characterisation

Following the request in early 2022 from SpF to French hospitals reporting grouped cases of SH bacteraemia, the FNRCS received 257 SH isolates obtained from neonates in 26 hospitals (n = 206 blood cultures or catheter, n = 22 carriage samples and n = 29 other types of samples) and 41 isolates either from the NICU environment (n = 37 from five NICUs) or from NICU staff (n = 4 from one NICU) between late 2021 to 2023. Ten of these hospitals also sent 41 SH isolates obtained from adult patients during the same period. Additionally, 157 SH isolates from patients in NICUs (neonates, n = 85) or non-NICUs (adults, n = 72) settings sent to the FNRCS for expertise prior to these reports of grouped cases i.e. between 2017 and 2021 were also included in the present study. All 496 isolates and sample attributes (year of isolation, isolation source, origin, geographical location, project and run accession numbers) are listed in Supplementary Table S1.

Isolates were grown on Columbia agar with 5% sheep blood (bioMérieux). Species assignation was confirmed by MALDI-TOF (Vitek MS system, bioMérieux). Antimicrobial susceptibility testing was performed on the automated Vitek2 system with AST-P631 or AST-P668 panels (bioMérieux) for 380 isolates. In addition, 48 isolates were tested using disc diffusion method and 10 tested using broth microdilution (Sensititre, ThermoFisher Scientific). For 58 isolates, antimicrobial susceptibility testing was not performed as part of or routine upon receipt and we have chosen not to analyse them retrospectively. Categorisation was interpreted according to the guidelines of the Antibiogram Committee of the French Society of Microbiology (CA-SFM; https://www.sfm-microbiologie.org/categories/sections-gt/comite-de-lantibiogramme) applicable at the time of analysis of the isolates, which are a French translation of the European Committee on Antimicrobial Susceptibility Testing (EUCAST) recommendations [16] with additional comments on technical aspects or on the interpretation of resistance mechanisms adapted to French practices (see the Supplement for interpretations that may affect the resistance percentages). Of note, Vitek2 results were also used to estimate vancomycin MIC as data provided by the manufacturer in the technical sheets indicate high essential agreement rates of ≥ 99.8%.

### Genome sequencing and genomic analysis

Whole genome sequencing (WGS) was performed for all 496 isolates and carried out in the genEPII department (Hospices Civils de Lyon, Lyon, France). DNA was extracted with the Maxwell RSC System (Promega) using the Maxwell RSC Blood DNA kit (Promega). Libraries were prepared using the DNA Prep library kit (Illumina) and sequencing was performed using either a MiSeq or a NextSeq 550 sequencer (Illumina) to obtain paired-end reads of 300 or 150 bp, respectively. Raw reads were trimmed for adapters and index removal. Trimmed reads were used to produce assemblies with SPAdes v3.14 [17]. Multilocus sequence typing (MLST) was performed using assemblies (https://github.com/tseemann/mlst). Identification of antibiotic resistance genes was performed using ABRicate (https://github.com/tseemann/abricate) and ResFinder v4.0 databases [18]. Virulence typing was performed using ABRicate software and an internal database that was constructed with previously described SH virulence associated genes [13,19,20].

Long-read sequencing using the RBK114–96 library preparation kit (Oxford Nanopore) and a R10.4.1 Flow Cell (Oxford Nanopore) was performed for a representative of the ST29 (ST20210379) clade in order to create a hybrid genome assembly with Illumina reads with close or almost close genomes and plasmids.

### Phylogeny

For comparison with international isolates, i.e. previously described NICU-associated SH clades, 199 SH isolates of known origin were included [9,14,21-23]. Isolates were chosen based on: (i) availability of raw data (fastq.gz), (ii) known geographical origin, (iii) known isolation source whenever it was available and (iv) date of isolation, whenever it was available. The year of isolation, isolation source, origin, geographical location, project and run accession numbers are provided in Supplementary Table S1.

Trimmed reads were aligned against the assembly of the SH reference isolate S167 (GenBank accession number: NZ_CP013911) and isolates for which the coverage of the reference genome sequence was lower than 70% or which had more than 25% missing data (gaps) were excluded. Variant calling and core genome SNP detection were performed using snippy v4.6.0 (https://github.com/tseemann/snippy). Positions with less than 10-fold sequencing depth and less than 90% unambiguous variant calls were excluded. SNPs resulting from recombination events were removed using gubbins v3.3 [24] and phylogenetic reconstruction was performed using IQTree with the GTR Gamma model [25]. The tree was rooted using a mid-point rooting.

In order to obtain a more refined view of each clade’s geographical distribution and niche specificity, a recombination-free SNP-based phylogeny was constructed for ST29 and ST25. Tree rooting was performed using a closely related isolate outside the clade (ST20172229 and ST20222371, respectively).

### Statistical analysis

Data processing and analysis were carried out using R software (version 3.6.1) or Stata version 10.0 software (StataCorp). For categorical variables, Pearson's chi-square test and Fisher’s exact pairwise test with Bonferroni correction method or Kruskal-Wallis tests were used. For continuous variables, Kruskal-Wallis tests followed by Dunn post-hoc tests with Bonferroni correction method were performed to compare groups or a Welch’s two-sample t-test (for normal distributions with unequal variances, SNP distances), Cohen’s kappa coefficient was used to assess the agreement between phenotypic and genotypic resistance [26]. All analyses were two-tailed and a p value ≤ 0.05 was considered significant.

## Results

**Figure 1:**
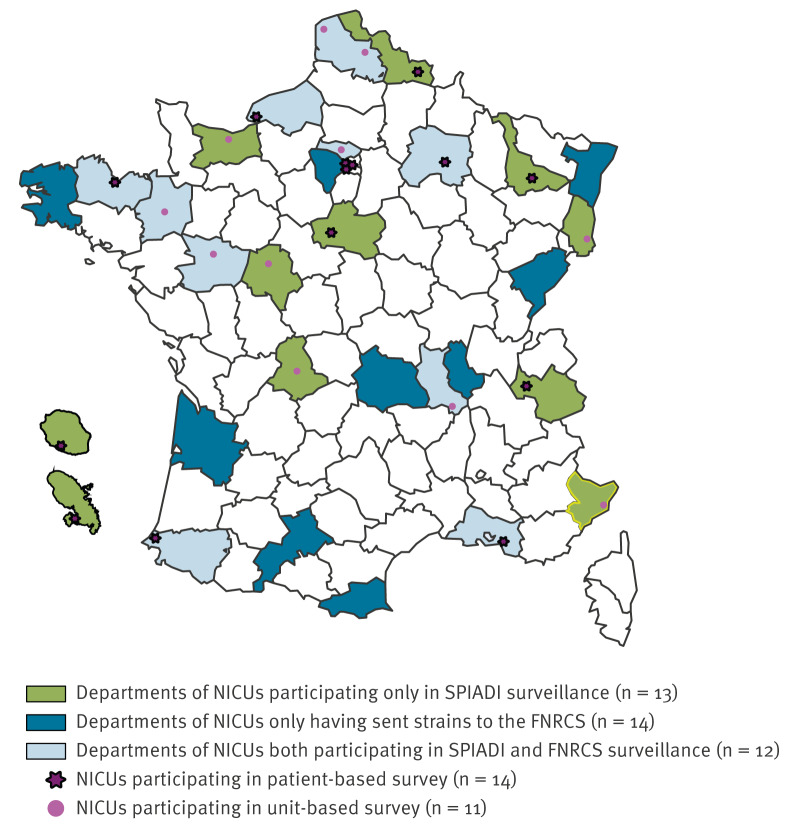
Geographical distribution of neonatal intensive care units participating in SPIADI surveillance of nosocomial bacteraemia or sending *Staphylococcus haemolyticus* strains to the French National Reference Centre for Staphylococci for analysis, France, 2019–2023 (n = 39)

FNRCS: French National Reference Centre for Staphylococci; NICU: neonatal intensive care units; SPIADI: National Network for Surveillance and Prevention of Infections Associated with Invasive Devices.

Our study included clinical data collected between 2019 and 2023 from 25 of the 67 French NICUs, which were selected because they had participated in at least four of the five SPIADI surveillance campaigns (Figure 1). Of these, 14 conducted a patient-based survey and 11 a unit-based survey. During the same period, 26 NICUs sent isolates to the FNRCS for epidemiological analysis, including 12 NICUs that participated in the SPIADI surveillance (n = 4 unit-based surveys and n = 8 patient-based surveys).

Geographical distribution of NICUs participating in the SPIADI network and NICUs that sent *Staphylococcus haemolyticus* isolates to the FNRCS for epidemiological assessment. The circles represent the approximate locations of the 11 NICUs participating in unit-based survey. The stars represent the approximate locations of the 14 NICUs participating in patient-based survey.

### Epidemiology of nosocomial bacteraemia in neonatal intensive care units from SPIADI surveillance 

Over the 5-year period, a total of 3,349 neonates were included in the 14 NICUs that conducted the patient-based survey. This surveillance revealed a population characterised by a median gestational age of 30–31 weeks of amenorrhoea including 56% (year 2019, 551/979) to 61% (year 2021, 362/595) of neonates with a birth weight ≤ 1,500 g (Table 1). We observed a shift between 2019 and 2020, with an increase in the percentage of neonates with a central venous catheter (CVC) from 74% to 82%, and a parallel decrease in the percentage of neonates with umbilical venous catheter (UVC) from 91% to 80%. These values seemed to stabilise from 2020 onwards.

**Table 1:** Characteristics of neonates included in SPIADI surveillance, France, 2019–2023

Characteristics	Year of survey^a^									
	2019		2020		2021		2022		2023	
	n	%	n	%	n	%	n	%	n	%
Neonates from 14 NICUs conducting patient-based surveys (n = 3,349)										
Total neonates	979	29	572	17	595	18	669	20	534	16
Sex ratio (M/F)	1.2		1.3		1.1		1.1		1.2	
Birth weight in g (median)	1,360		1,382		1,285		1,360		1,385	
Neonates ≤ 1,500 g	551	56	318	56	362	61	382	57	302	57
Neonates ≤ 750 g	98	10	63	11	69	12	67	10	69	13
Gestational age (median WA)	31		31		30		31		31	
Neonates < 25 WA	43	4	19	3	30	5	18	3	27	5
Death during hospitalisation	95	10	40	7	46	8	57	9	45	8
**Neonates with CVC**										
Total	722	74	467	82	496	83	562	84	455	85
CVC ratio^b^	1.5		2.1		2.4		2.3		2.2	
Catheterisation duration (median days)	9		10		10		10		11	
Use for lipids	654	91	414	89	466	94	530	94	428	94
CVC-related NB (per 100 CVC)	78	11	64	14	42	8	40	7	27	6
**Neonates with UVC**										
Total	888	91	455	80	461	77	507	76	414	77
UVC ratio^b^	8.2		3.8		3.2		3.0		3.0	
Catheterisation duration (median days)	3		4		3		3		4	
Use for lipids	569	64	324	71	339	74	393	78	323	78
UVC-related NB (per 100 UVC)	24	3	6	1	10	2	5	1	7	2
Neonates with NB in 25 NICUs (n = 474)										
Sex ratio (M/F)	1.5		1.2		1.0		1.9		1.7	
Birth weight in g (median)	970		958		840		900		995	
Neonates ≤ 1,500 g	57	69	59	76	94	90	96	81	67	74
Neonates ≤ 750 g	21	25	19	24	33	32	40	34	27	30
Gestational age (median WA)	28		28		27		27		27.5	
< 25 WA (%)	7	8	5	6	13	13	7	6	12	13
Death within 7 days after the start of NB	8	10	6	8	10	10	10	8	9	10
NB episodes	83	18	78	16	104	22	119	25	90	19
*S. aureus*	14	17	7	9	14	13	15	13	11	12
*S. epidermidis *	30	36	32	41	43	41	39	33	33	37
*S. haemolyticus *	15	18	17	22	31	30	37	31	24	27
*S. capitis*	10	12	8	10	11	11	15	13	7	8
Other species	20	24	24	31	26	25	35	29	28	31
**NB incidence rates (per 1,000 PDs)**										
All NB	4.3		3.2		3.6		4.7		4.0	
*S. aureus*	1.6		0.4		0.5		0.4		1.0	
*S. epidermidis*	1.6		1.1		1.3		1.4		1.5	
*S. haemolyticus*	0.6		0.7		1.0		1.2		1.2	
*S. capitis*	0.3		0.5		0.4		0.6		0.3	

CVC: central venous catheter; NB: nosocomial bacteraemia; NICU: neonatal intensive care unit; PDs: patient-days; UVC: umbilical venous catheter; WA: weeks of amenorrhoea.

^a^ The 3-month period survey occurred annually between 1 January and 15 July.

^b^ The CVC or UVC ratio is calculated as neonates with/without CVC or UVC, respectively.

During the 5-year period, 474 neonates in the 25 participating NICUs presented with nosocomial bacteraemia (78–119/year, Table 1). The primary source of nosocomial bacteraemia was an intravascular catheter (n = 248; 52%). Information on the characteristics of the 474 nosocomial bacteraemia acquired in the 25 NICUs between 2019 and 2023, including source of nosocomial bacteraemia, duration of the nosocomial bacteraemia and identified microorganisms, is provided in Supplementary Table S2. Coagulase-negative staphylococci (CoNS) represented 67% (365/546) of the microorganisms associated with nosocomial bacteraemia. The neonates affected by SH or *S. capitis* nosocomial bacteraemia had a lower birth weight (93% (115/124) and 92% (47/51) with birth weight ≤ 1,500 g, respectively) vs neonates with nosocomial bacteraemia caused by *S. aureus* or *S. epidermidis* (69% (42/61) and 71% (126/177), respectively; Chi-square test between the four sub-populations, p < 0.001). Notably, although not statistically significant, SH was the only pathogen for which the proportion among nosocomial bacteraemia has shown an increase from 18% in 2019 to a maximum of 31% in 2022. The incidence rate of nosocomial bacteraemia varied from 3.2 NB/1,000 patient days (PDs) in 2020 to a maximum of 4.7 NB/1,000 PDs in 2022 (Kruskal-Wallis test, p = 0.46). The SH nosocomial bacteraemia increased slightly, from 0.6/1,000 PDs in 2019 to a maximum of 1.2/1,000 PDs in 2022 and 2023 (p = 0.70).

### Genetic diversity, antibiotic resistance and virulence factors of *Staphylococcus haemolyticus* isolates

**Figure 2:**
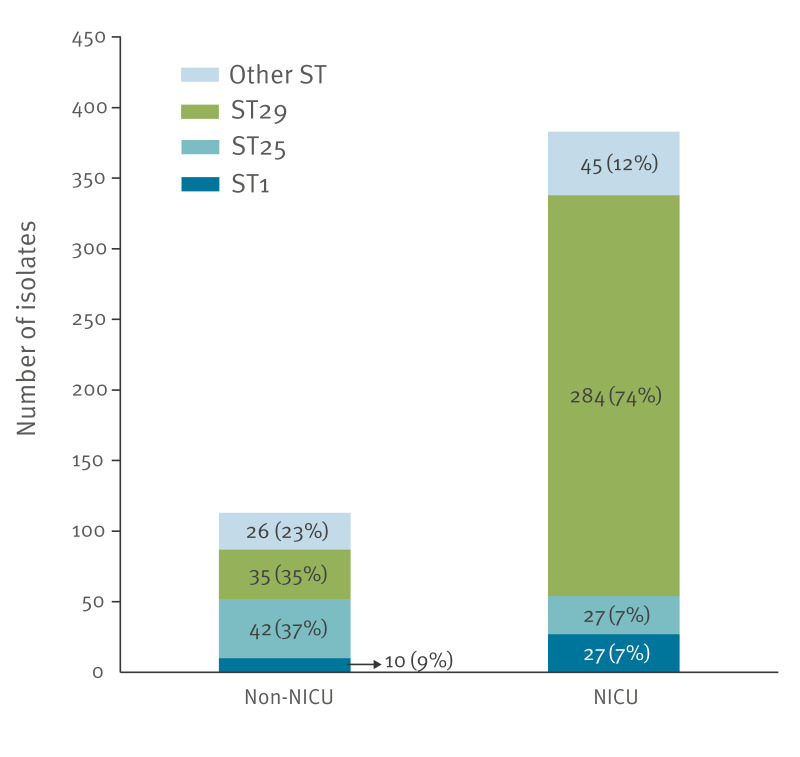
Multilocus sequence typing diversity of *Staphylococcus haemolyticus* isolates associated with nosocomial infections sent to the French National Reference Centre for Staphylococci, France, 2017–2023 (n = 496)

NICU: neonatal intensive care unit; ST: sequence type.

The diversity of genetic backgrounds of 496 *S. haemolyticus* isolates from the NICU (n = 342 from neonates, n = 37 from environment and n = 4 from staff) or non-NICU (n = 113) settings between 2017 and 2023 was assessed by multilocus sequence typing at the FNRCS. They were assigned into 19 different sequence types with 96% (474/496) of the isolates grouping in clonal complex CC3. Of these, 1% (9/474) belonged to the reference ST of CC3, namely ST3, 82% (387/474) or 16% (78/474) belonged to STs that were respectively single or double locus variants of ST3. ST29 (single locus variant of ST3) was predominant, accounting for 64% (319/496) of all isolates (Figure 2). The genetic background determined by MLST of all SH isolates is provided in Supplementary Table S3. ST25 (double locus variant of ST3) and ST1 (single locus variant of ST3) were the two other most frequent STs, representing 14% (69/496) and 8% (37/496) of isolates, respectively. Although this pattern was observed for both NICU and non-NICU isolates, ST29 was particularly pervasive in the NICU setting, accounting for 74% (284/383) of NICU isolates but only 31% (35/113) of non-NICU isolates. Conversely, ST25 isolates were found more frequently in non-NICU settings (37%, 42/113) than in NICUs (7%, 27/383); ST1 isolates were found at similar levels in non-NICU (9%, 10/113) and NICU settings (7%, 27/383).

Analyses were performed at the French National Reference Centre for Staphylococci on 496 isolates collected between 2017 and 2023 from the NICU (n = 342 from neonates, n = 37 from environment and n = 4 from staff) or non-NICU (n = 113) settings. For each ST group presented, the number of isolates and their frequency (%) are indicated in the bars for NICU and non-NICU settings. Among ST29 isolates (n = 319), 252 were from neonates, 32 were from NICU environment or staff, 35 were from adults in non-NICU-settings. Numbers and percentages of isolates belonging to each sequence type (ST) are indicated. Other STs are listed in Supplementary Table S3.

All French SH isolates showed a general MDR profile. However, ST29, ST25 and ST1 isolates showed higher resistance rates than those of other sequence types, notably for gentamicin and quinolones (Fisher’s exact pairwise test, p < 0.001) (Table 2). The ST29 and ST25 isolates also exhibited significantly higher resistance rates to oxacillin (p < 0.001 and p = 0.035, respectively) and to cotrimoxazole (p < 0.001) compared with other sequence types, with over 94% of isolates exhibiting resistance. ST29 isolates were also more frequently resistant to clindamycin and rifampicin (p < 0.001), with over 99% isolates resistant. The simultaneous resistance to oxacillin, gentamicin, quinolones, cotrimoxazole, clindamycin and rifampicin was a characteristic of ST29 with a specificity of 99% (275 ST29 isolates of the 278 isolates with such resistance profile) and a sensitivity of 93% (275 ST29 isolates with this multidrug resistance profile among the 296 ST29 isolates for which the antibiogram was available). Many SH isolates showed MICs near or above the breakpoint for vancomycin (30% of isolates with Vitek 2 estimated MIC ≥ 2 mg/L). 

**Table 2:** Phenotypic resistance to the main antibiotic classes and distribution of resistance-associated genes in *Staphylococcus haemolyticus* isolates, France, 2017–2023 (n = 438)

ST	**Isolate characteristics **	**Oxacillin**	**Gentamicin**	**Erythromycin**	**Clindamycin**	**Tetracycline**	**Quinolones^a^**	**Pristinamycin**	**Linezolid**	**Cotrimoxazole**	**Rifampicin**	**Fosfomycin**	**Fusidic acid**	**Vancomycin**	**Mupirocin**
ST1	% resistant	94	97	91	15	45	97	13	3	42	79	100	30	0	NT
	n resistant/n tested	31/33	32/33	30/33	5/33	13/29	32/33	4/30	1/33	14/33	26/33	27/27	10/33	0/28	NT
	Resistance gene(s)	*mec*A	*aac (6 ´)-le-aph (2 ´ ´); aph(3′)IIIa; ant(4’)-Ia*	*msrA; mphC; ermC; isaB*	*vga(A)LC*	*tetK*	NT	NF	*cfr*	*dfrG*	*rpoB mutations* ^c^	*fosD*	*fusB; fusC*	NF	*mupA*
	% resistance genes	94	97; 45; 9	97; 97; 15; 3	6	3	NA	NA	3	39	73	3	27; 3	NA	3
	Genotype/phenotype agreement^b^	1	1	0.48	0.53	0	NA	NA	1	0.81	0.82	0	1	NA	NA
ST25	% resistant	97	98	97	33	7	98	0	0	97	5	95	5	0	NT
	n resistant/n tested	56/58	57/58	56/58	19/58	3/42	57/58	0/45	0/58	56/58	4/58	36/38	3/58	0/41	NT
	Resistance gene(s)	*mec*A	*aac (6 ´)-le-aph (2 ´ ´); aph(3′)IIIa; ant(4’)-Ia*	*msrA; mphC; ermC*	*vga(A)LC*	*tetK*	NT	NF	None	*dfrG*	*rpoB mutations* ^c^	*fosB6*	*fusC*	NF	*mupA*
	% resistance genes	97	98; 88; 2	98; 98; 40	2	2	NA	NA	NA	98	6	2	3	NA	3
	Genotype/phenotype agreement^b^	1	1	0.66	0.07	0.48	NA	NA	NA	0.66	0.85	0	0.79	NA	NA
ST29	% resistant	100	100	100	99	13	100	14	0.3	94	100	88	0.7	0.4	NT
	n resistant/n tested	296/296	296/296	295/296	294/296	39/295	295/296	30/218	1/286	278/296	296/296	247/281	2/293	1/257	NT
	Resistance gene(s)	*mec*A	*aac (6 ´)-le-aph (2 ´ ´); aph(3′)IIIa; ant(4’)-Ia*	*msrA; mphC; ermC; isaB*	*vga(A)LC*	*tetK*	NT	NF	*cfr*	*dfrG; dfrC*	*rpoB mutations* ^c^	*fosB1; fosB6; fosD*	*fusB*	NF	*mupA*
	% resistance genes	100	100; 98; 82	100; 100; 3; 0.3	99	0.3	NA	NA	0.3	94; 1	99	8; 0.3; 0.3	1	NA	95
	Genotype/phenotype agreement^b^	1	1	1	0.66	0.04	NA	NA	1	0.97	1	0.2	0.4	NA	NA
Other STs	% resistant	78	57	81	18	33	57	2	0	28	28	97	33	3	NT
	n resistant/n tested	40/51	29/51	41/51	9/51	14/42	29/51	1/47	0/51	14/51	14/50	33/34	17/51	1/35	NT
	Resistance gene(s)	*mec*A	*aac (6 ´)-le-aph (2 ´ ´); aph(3′)IIIa; ant(4’)-Ia; ant(9)-Ia*	*msrA; mphC; ermC; ermA; erm(45)*	*vga(A)L; vgaA; lnuA*	*tetK; tetL; tetM*	NT	NF	None	*dfrG*	*rpoB mutations* ^c^	*fosD*	*fusB; fusC*	NF	*mupA*
	% resistance genes	78	65; 49; 10; 2	73; 73; 12; 2; 2	12; 31; 2	4; 4; 2	NA	NA	NA	27	18	2	22; 8	NA	3
	Genotype/phenotype agreement^b^	1	0.62	0.81	0.35	0.35	NA	NA	NA	0.9	0.91	0	0.91	NA	NA

NA: not applicable; NF: not found; NT: not tested; ST: sequence type.

^a^ Ofloxacin or levofloxacin were tested in accordance with the recommendations of the Antibiogram Committee of the French Society of Microbiology (CA-SFM; https://www.sfm-microbiologie.org/categories/sections-gt/comite-de-lantibiogramme) at the time of arrival of the isolate.

^b^ The agreement between the presence of resistance genes and phenotypic resistance, as expressed by the kappa coefficient, can be interpreted as follows: values ≤ 0 as indicating no agreement, 0.01–0.20 as none to slight, 0.21–0.40 as fair, 0.41–0.60 as moderate, 0.61–0.80 as substantial and 0.81–1.00 as almost perfect agreement.

^c^ The *rpoB* non-synonymous mutations detected and associated with rifampicin resistance were D471E, I527M and S532N.

We observed good correlation between the presence of resistance genes and the phenotype. ST29 isolates harboured more antibiotic resistance genes than isolates from other sequence types, including the *vga(A)*-LC gene (p < 0.001), associated with resistance to clindamycin. Triple non-synonymous mutations in the *rpoB* gene (D471E, I527M and S532N), which has been previously reported to confer rifampicin resistance in SH [27], were identified in 99% of ST29 strains but rarely in other STs (Fisher’s exact pairwise test, p < 0.001). Finally, the *mupA* gene encoding mupirocin resistance was found in 95% of ST29 isolates but in less than 3% of other isolates (p < 0.001).

No major differences were observed in the virulence repertoire of the different genetic backgrounds. Genes encoding for adhesion factors such as the serine rich surface protein SraP, biofilm synthesis, or cytolytic toxins, such as the alpha-haemolysin or PSM alpha and beta1 were detected in 99 to 100% of the French SH isolates, indistinctively of the genetic background (ST). Other virulence genes previously associated with nosocomial infections [13] were also widely present in the French SH collection. The complete list of virulence factors and their distribution is provided in Supplementary Table S4.

### Phylogenetic analysis of *Staphylococcus haemolyticus* French isolates

**Figure 3:**
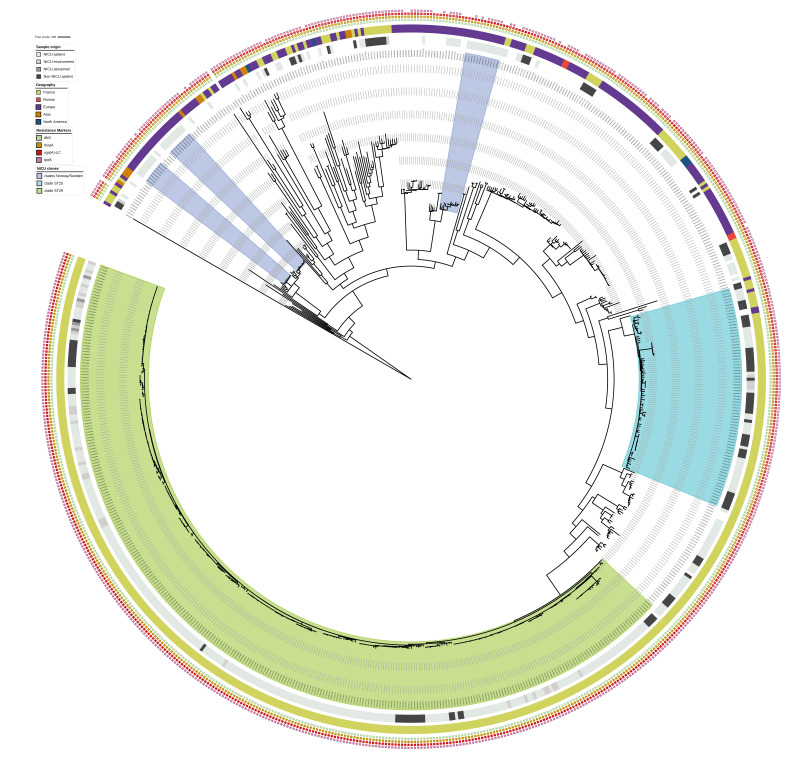
Phylogeography of French (n = 490) and international *Staphylococcus haemolyticus* isolates (n = 199), 1988–2023

NICU: neonatal intensive care unit; ST: sequence type.

All 496 French SH isolates were compared with 199 previously described SH isolates of known origin, including isolates from SH NICU clades detected in Northern Europe [9,14]. Six FNRCS isolates were excluded because of a very high divergence or wrong species assignment. The phylogenetic analysis revealed the presence of two main clades found in France, which we called the ST29 and ST25 clades, with the remaining French isolates being interspersed between these two clades and international isolates (Figure 3). Importantly, these two new ST25 and ST29 clades were distinct from the three clusters of SH infections previously reported in Norwegian or Swedish NICUs [9,14].

Midpoint rooting maximum likelihood phylogeny was constructed based on SNPs in the core genome obtained after removal of recombination events. The outer rings indicate the presence of resistance genetic markers frequently found in ST1, ST25 and ST29 isolates (*dfrG, mupA, vga(A)-LC* genes and *rpoB* point mutations associated with rifampicin resistance). When information was available: presence of resistance markers is represented by full coloured squares and absence by empty squares. Missing squares indicate that the information could not be interpreted (insufficient coverage of the gene). The solid multicolour ring indicates the geographical origin; the black/grey ring indicates the sample origin (when available). Among French isolates, 342 were from neonates, 38 were from NICU environment or staff, and 110 were from adults in non-NICU-settings. Clades corresponding to clonal populations previously identified in Norwegian or Swedish NICUs are shaded in light purple and clades corresponding to the two French specific clades are shaded in light blue (ST25 clade) and in light green (ST29 clade).

The main ST29 clade grouped 314 of 319 isolates belonging to ST29, which were isolated either inside (n = 283) or outside (n = 31) the NICU setting. As described above, this clade was characterised by the presence of the *mupA* and *vga(A)-LC* genes. Importantly, a hybrid assembly of short and long reads showed that this lineage carried two plasmids, both of which harboured resistance markers: the pSH1275–3 plasmid (GenBank: CP123982.1) harbouring the *vga(A)*-LC gene and a previously unknown plasmid of 33.6 kb harbouring the *mupA* and the *ant(4’)-Ia* gene (coding for aminoglycosides resistance).

**Figure 4:**
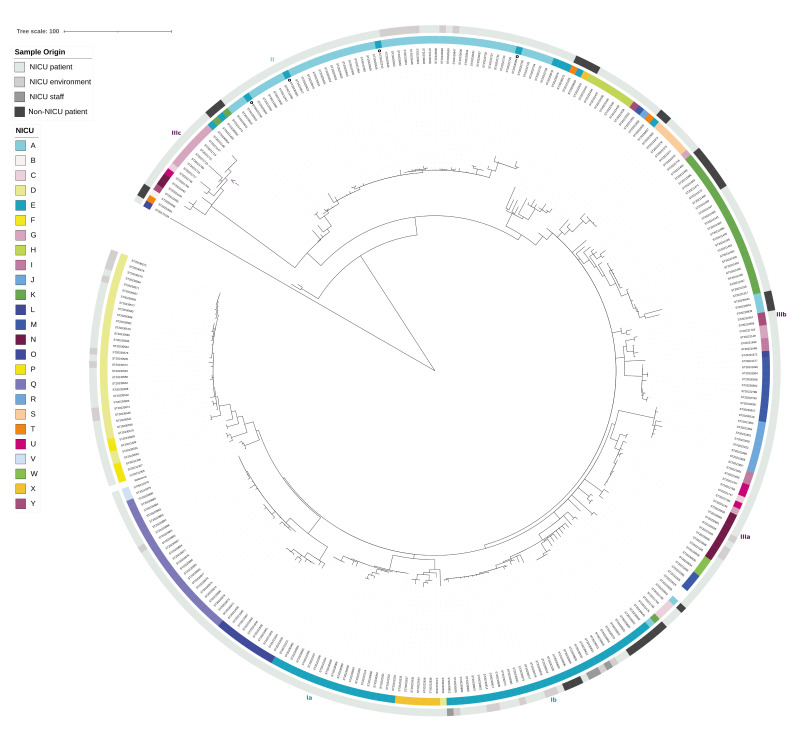
Phylogeography of the *Staphylococcus haemolyticus* ST29 clade, France, 2017–2023 (n = 319)

NICU: neonatal intensive care unit; ST: sequence type.

The ST29 phylogeny illustrates the spread of this clonal population throughout mainland France, with isolates found in 25 of the 26 NICUs that sent isolates to the FNRCS (Figure 4).

Rooted maximum likelihood phylogeny was constructed based on SNPs in the core genome obtained after removal of recombination events. The outer black/grey ring indicates the sample origin. Among ST29 isolates (n = 319), 252 were from neonates, 32 were from NICU environment or staff and 35 were from adults in non-NICU-settings. The inner multicolour ring refers to the geographical origin with each NICU (one NICU per city) identified by a different colour. For NICUs within the region of Île-de-France, encompassing nearby cities (NICUs C, G, I, N, U, Y), a gradient of pink gradient colours was used. Three groups of isolates are highlighted by Roman numbering I to III. Isolates from the NICU E grouped in three populations, Ia, Ib and of six isolates clustering with isolates of NICU A (group II). Transfer of neonates between these NICUs were confirmed for four of them indicated with a black circle between the isolate name and the isolation origin circle. Isolates from the region of Île-de-France grouped in three populations (IIIa, IIIb and IIIc). A recombinant clade is indicated by a pink arrow.

Furthermore, this clonal population seems to have evolved in each NICU as isolates from the same NICU (including isolates from neonates, environment and staff; median distance between isolates was 7 SNPs with standard deviation (SD) of 17) were generally more closely related with each other than with isolates from other NICUs (Welch t-test, p < 0.001; median distance of isolates from different NICUs was 66 SNP with SD of 32). However, we did not detect potential adaptative mutations or enrichment in mobile genetic elements that could explain a local adaptation to each NICU. Interestingly, two distinct populations were identified in one of the NICUs (NICU E; Figure 4), with one population (Figure 4, group Ia) found only in neonates, and the second population (Figure 4, group Ib) found more widely within the NICU (including neonates, environment and staff) and outside the NICU. In addition, six isolates from this NICU were clustered within the group of isolates from a neighbouring city (NICU A; Figure 4, group II). For four of the isolates, transfers of neonates between NICUs A and E were confirmed (Figure 4, isolates highlighted by a black circle in group II). Two striking features were observed for the group of NICUs in the Île-de-France (Paris Metropolitan) region with three distinct sub-clades (Figure 4, group III a, b and c which are composed of NICUs coloured with a rose/pink gradient). On one hand, all three sub-clades include isolates from multiple NICUs of the Île-de-France region. However, no infant transfer of neonates could be confirmed between these NICUs, limiting the interpretation of the distribution of isolates from these NICUs around the phylogenetic tree. On the other hand, sub-clade IIIa (Figure 4) comprises divergent isolates showing more recombination events than the remaining ST29 isolates. The phylogenetic tree obtained after removal of recombination events and indications of the positions of recombination events in the genome are provided in Supplementary Figure S1.

**Figure 5:**
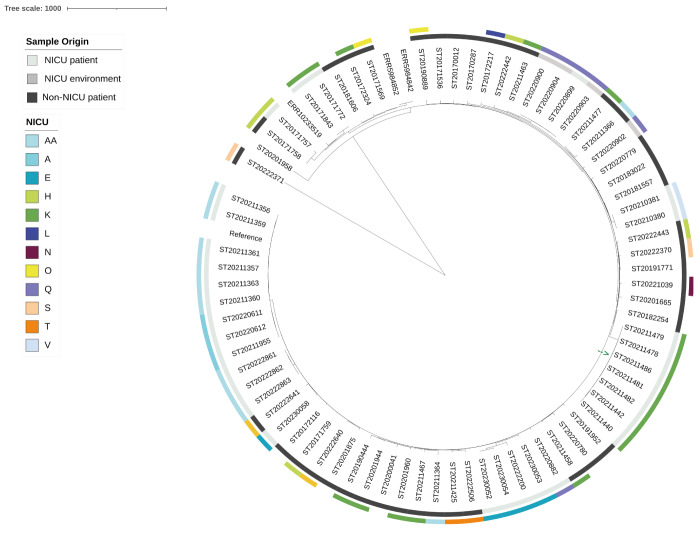
Phylogeography of the *Staphylococcus haemolyticus* ST25 clade, France, 2017–2023 (n = 73)

NICU: neonatal intensive care unit; ST: sequence type.

The ST25 clade was comprised of 73 French isolates from either inside (n = 35) or outside (n = 38) the NICU setting, and three European isolates [14,21] (Figure 5). It included 65 of the 69 French ST25 isolates, three European ST25 isolates but also eight of the 37 French ST1 isolates. Indeed, seven of these eight isolates corresponded to a sub-clade of isolates showing multiple recombination events, including one recombination event resulting in a distinct sequence type as ST1 presents only one-allele variation from ST25. The phylogenetic tree obtained after removal of recombination events and indications of the positions of recombination events in the genome are provided in Supplementary Figure S2. Compared to clade ST29, the ST25 clade demonstrated a less pronounced tropism for NICUs as it was identified in only six of the 26 NICUs. Nonetheless, ST29 was not detected in one of these six NICUs (Figure 5, NICU AA). 

Rooted maximum likelihood phylogeny was constructed based on SNPs in the core genome obtained after removal of recombination events. The outer multicolour ring indicates the geographical origin with each NICU (1 NICU per city) identified by a different colour. The inner black/grey ring indicates the sample origin. There were 73 French isolates in the ST25 clade; 31 were from neonates, 4 were from NICU environment, 38 were from adults in non-NICU-settings. A recombinant clade is indicated by a green arrow.

## Discussion

Following an increase in the number of grouped cases of SH-associated infections in French NICUs since late 2021, we combined data from the national surveillance SPIADI network with phenotypic and genotypic data from a large collection of SH hospital-associated isolates sent to the FNRCS. This approach allowed the assessment of SH infections in neonates from 2019 to 2023 and examination of the molecular epidemiology to characterise potential SH clades associated with bacteraemia in neonates. 

The proportion of SH among cases of nosocomial bacteraemia increased from around 20% to 30% within the 5-year period, indicating that it is the second most common pathogen alongside *S. epidermidis* that mainly affects neonates with very low birth weight.

Genomic analysis of a comprehensive collection of 496 French SH isolates compared with 199 international isolates, including isolates from known SH NICU clades [9,14,21,22], revealed the predominance of the ST29 and, to a smaller extent, the ST25 lineage in French NICUs. Both ST25 and ST29 belong to the highly prevalent clonal complex CC3 [4], which includes other dominant clones from ST1 [5,9] and ST42 [8], and was previously classified in the CC29. However, previous studies have reported very low prevalence for either ST25 or ST29 [4,5,9]. Moreover, both French clonal lineages are distinct from clonal expansions outlined in prior studies [8,9,14], which could suggest that SH clades are not able to successfully spread as widely as the *S. capitis* NRCS-A clade, whose diffusion in NICUs was reported worldwide in the last decade [28].

Although the exact reason for the epidemiological success of ST29 remains unknown, its highly resistant profile is undoubtedly a strong driver. Indeed, this clade was characterised by multidrug resistance to at least six classes of antibiotics, including resistance to clindamycin and rifampicin. This phenotypic feature distinguished this ST from other prevalent multidrug-resistant sequence types identified in our study (ST1 and ST25) with a good sensitivity of 93% and specificity of 99%, suggesting that the resistance profile could be used as a screening marker in routine use for infection control interventions. The presence of RpoB triple point mutations (D471E, I527M and S532N) in this clade is of concern, as two of these mutations (D471E and I527M) have previously been associated with crossed hetero-resistance to vancomycin in successful globally disseminated MDR *S. epidermidis* nosocomial clades [29], but also in SH isolates [27]. Although we have not studied vancomycin hetero-resistance in our isolate collection, the impact of these dual RpoB mutations on vancomycin MICs has been shown to be variable between strains [27,29]. Nonetheless, as vancomycin is an important first-line antibiotic treatment in neonates, these mutations may have contributed to the selection of the ST29 clade. 

ST29 isolates were also characterised by the presence of plasmid-born *mupA* and *vga(A)LC* genes, in distinct plasmids. The prevalence of the latter plasmid in the ST29 clade is surprising as clindamycin is not commonly prescribed in NICUs and this small plasmid did not contain any other resistance marker. The high prevalence of the *mupA* gene is another point of concern, as it represents a potential risk of horizontal transfer to other SH sequence types or *S. aureus* nosocomial lineages [30,31]. This could affect the ability to use mupirocin for decolonisation procedures in *S. aureus* carriers to prevent infections.

Our data suggest that the clonal ST29 population, once it has entered the NICU, is able to evolve in the environment of each NICU, as the genetic distance between strains from a given NICU was smaller than the distance between strains from different NICUs. As described in previous studies [5,6,13,32], numerous recombination events were detected in ST29 and ST25 lineages, contributing to the evolution of SH.

We recognise limitations of our study. The bias in the FNRCS collection, presented by the small number of isolates from 2017 to 2021 compared to the artificially large number of isolates corresponding to grouped cases reported in late 2021 and 2022 to SpF, did not allow us to distinguish between ongoing outbreaks or the identification of endemic. 

Following SpF’s alert on the rise of SH infections in NICUs, the French learned societies of microbiology, neonatal medicine and infection prevention were urged by the French Ministry of Health to make specific recommendations to improve their diagnosis, treatment and prevention [33]. Of note, after an increase in the incidence of SH infections from 2019 to 2022, it seemed to stabilise in 2023, a phenomenon that should be monitored in the coming years. We believe that this stabilisation may be partly explained by the infection control measures implemented in NICUs after the publication of these recommendations although this was not evaluated in the present study.

## Conclusions

We detected the predominance of a successful and highly resistant lineage that have disseminated in French NICUs, the ST29 clade, mainly affecting very low birth weight premature neonates. This study highlights the importance of the surveillance of SH infections in NICUs to better understand and anticipate epidemics, and an extension of this surveillance should be envisaged to other European countries.

## Supplementary Data

385000 Supplement

## Supplementary Data

65000 Supplementary_Table_S1
