# Supplementary material for: Epidemiology of Staphylococcus haemolyticus nosocomial bacteraemia in neonatal intensive care units, France, 2019 to 2023: predominance of the ST29 (CC3) multidrug-resistant lineage
Source: Euro Surveill. 2025 Mar 20;30(11):2400309. doi: 10.2807/1560-7917.ES.2025.30.11.2400309 (PMC11927072; doi:10.2807/1560-7917.ES.2025.30.11.2400309)
Supplement: Supplement [file 24-00309_SIMOES_Supplement.pdf]

Title: Epidemiology of *Staphylococcus haemolyticus* nosocomial bloodstream infections in French neonatal intensive care units (2019-2023): predominance of the ST29 (CC3) multidrug-resistant lineage

This supplementary material is hosted by *Eurosurveillance* as supporting information alongside the article "Epidemiology of *Staphylococcus haemolyticus* nosocomial bloodstream infections in French neonatal intensive care units (2019-2023): predominance of the ST29 (CC3) multidrug-resistant lineage", on behalf of the authors, who remain responsible for the accuracy and appropriateness of the content. The same standards for ethics, copyright, attributions and permissions as for the article apply. Supplements are not edited by *Eurosurveillance* and the journal is not responsible for the maintenance of any links or email addresses provided therein.

Supplementary material concerning the Material and Methods section:

Staphylococcus haemolyticus isolates and phenotypic characterisation

The guidelines of the Antibiogram Committee of the French Society of Microbiology are a French translation of the European Committee on Antimicrobial Susceptibility Testing (EUCAST) recommendations with additional comments on technical aspects or on the interpretation of resistance mechanisms adapted to French practices. For the present study, interpretations that may affect the resistance percentages presented are: i) clindamycin: EUCAST recommends to classify clindamycin as resistant if even if it classified as susceptible according to the minimum inhibitory concentration (MIC) in case there is an inducible MLSb mechanism. On the contrary, CASFM recommends to classify it only according to the MIC values, regardless of resistance mechanisms; ii) vancomycin: EUCAST recommends a clinical breakpoint of 4 for coagulase negative staphylococci, whereas CASFM recommends a clinical breakpoint of 2.

Supplementary Table S1. List of SH strains used in this study

Excel file

Supplementary Table S2. Characteristics of the 474 NB acquired in the 25 NICUs (2019-2023).

Survey campaigns	2019	2020	2021	2022	2023	2019-2023
NB source, n	83	78	104	119	90	474
Intravascular devices	36	40	54	65	53	248
Pulmonary	9	7	5	5	5	31
Urinary	1	1	2	2	1	7
Digestive/abdominal	10	4	9	10	10	43
Other sources	11	13	4	8	8	44
No source found	16	13	30	28	13	101
Duration between the beginning of NB and entry (median value ; days)	10	11	10	8	11	
Microorganisms identified, n	89	88	125	141	103	546
Staphylococci						
S. aureus (per 100 NB)	14 (16.9)	7 (9.0)	14 (13.5)	15 (12.6)	11 (12,2)	61 (12.9)
S. epidermidis (per 100 NB)	30 (36.1)	32 (41.0)	43 (41.3)	39 (27.7)	33 (31.7)	177 (37.3)
S. haemolyticus (per 100 NB)	15 (18.1)	17 (21.8)	31 (29.8)	37 (31.1)	24 (26.7)	124 (26.2)
S. capitis (per 100 NB)	10 (12.0)	8 (10.2)	11 (10.6)	15 (12.6)	7 (8.7)	51 (10.8)
Other coagulase-negative staphylococci	2	1	5	3	2	13
Enterococcus sp	4	3	4	6	4	21
Streptococcus agalactiae	0	2	0	1	0	3
Bacillus cereus	2	1	1	2	1	7
Enterobacterales (per 100 NB)	10 (12.0)	14 (17.9)	11 (10.6)	16 (13.4)	11 (12.2)	62 (13.1)
E. coli	2	5	5	5	3	20
Enterobacter sp	3	5	3	1	4	16
Klebsiella sp	4	2	2	7	3	18
others	1	2	1	3	1	8
Pseudomonas aeruginosa	0	2	1	2	0	5
Acinetobacter sp	0	0	0	1	2	3
Achromobacter sp	0	0	0	0	5	5
Candida sp	1	0	4	1	3	9
Others	1	1	0	3	0	5

NB: nosocomial bacteraemia; CVC: central venous catheters (all peripherally inserted central catheter (PICC) and centrally inserted central catheter (CICC); UVC: umbilical venous catheter; WA: weeks of amenorrhea;

Supplementary Table S3. Genetic background of SH isolates sent to the FNRCS from January 2017 to March 2023, as assessed by Multi Locus sequencing Typing (MLST).

MLST	origin	2017-2021	2022-2023	Clonal Complex
**ST1**	NICU	19	8	CC3 (single locus*)
	non NICU	9	1	
**ST2**	NICU	1	0	CC3 (single locus*)
	non NICU	0	0	
**ST25**	NICU	12	15	CC3 (double locus*)
	non NICU	31	11	
**ST29**	NICU	31	253	CC3 (single locus*)
	non NICU	14	21	
**ST3**	NICU	4	3	CC3 (reference ST)
	non NICU	3	0	
**ST40**	NICU	0	2	CC3 (single locus*)
	non NICU	0	0	
**ST42**	NICU	12	0	CC3 (single locus)
	non NICU	1	0	
**ST49**	NICU	0	3	CC3 (single locus*)
	non NICU	0	0	
**ST52**	NICU	0	3	
	non NICU	3	4	
**ST53**	NICU	0	0	
	non NICU	0	1	
**ST54**	NICU	0	1	
	non NICU	0	0	
**ST56**	NICU	0	1	CC3 (double locus*)
	non NICU	1	1	
**ST65**	NICU	0	0	CC3 (double locus*)
	non NICU	3	0	
**ST69**	NICU	6	0	CC3 (single locus*)
	non NICU	4	0	
**ST8**	NICU	0	1	CC3 (double locus*)
	non NICU	1	0	
**ST9**	NICU	0	0	
	non NICU	1	0	
**ST104**	NICU	0	1	
	non NICU	0	0	
**ST115**	NICU	0	2	
	non NICU	0	0	
**new ST**	NICU	0	5	CC3 (1 strain is a double locus and 2 strains are single locus*)
	non NICU	1	2	

***single and double locus variants of ST3**

Supplementary Table S4. Distribution of virulence genes in SH isolates.

	ST1	ST25	ST29	other ST
**PSMalpha**	37(100%)	69(100%)	319(100%)	70 (99%)
**PSMbeta1**	37(100%)	69(100%)	319(100%)	71 (100%)
**alpha-hemolysin**	37(100%)	69(100%)	319(100%)	71 (100%)
**sraP**	37(100%)	67(97%)	319(100%)	63 (89%)
**nikA**	37(100%)	69(100%)	319(100%)	54 (76%)
**secA2**	37(100%)	67(97%)	319(100%)	56 (78%)
**fabG**	37(100%)	69(100%)	319(100%)	63 (89%)
**MFS_SH1055**	37(100%)	69(100%)	319(100%)	65 (92%)
**yodJ**	37(100%)	69(100%)	319(100%)	70 (99%)
**ansA**	37(100%)	69(100%)	319(100%)	71 (100%)
**SH1612**	36(97%)	68(99%)	316(,99%)	46 (65%)
**SH2156**	37(100%)	69(100%)	319(100%)	66 (93%)
**folB**	37(100%)	69(100%)	319(100%)	71 (100%)
**folP**	35(95%)	65(94%)	318(100%)	42 (59%)
**SH2607**	23(62%)	68(99%)	310(97%)	39 (55%)
**ydaF**	36(97%)	68(99%)	313(98%)	46 (65%)

The number and frequences in brackets of positive isolates are indicated.

Figure S1. French ST29 clonal population rooted Maximum Likelihood phylogeny based on substitutions in the core genome obtained after removal of recombination events and recombination map. The recombinant clade is indicated by the "*".

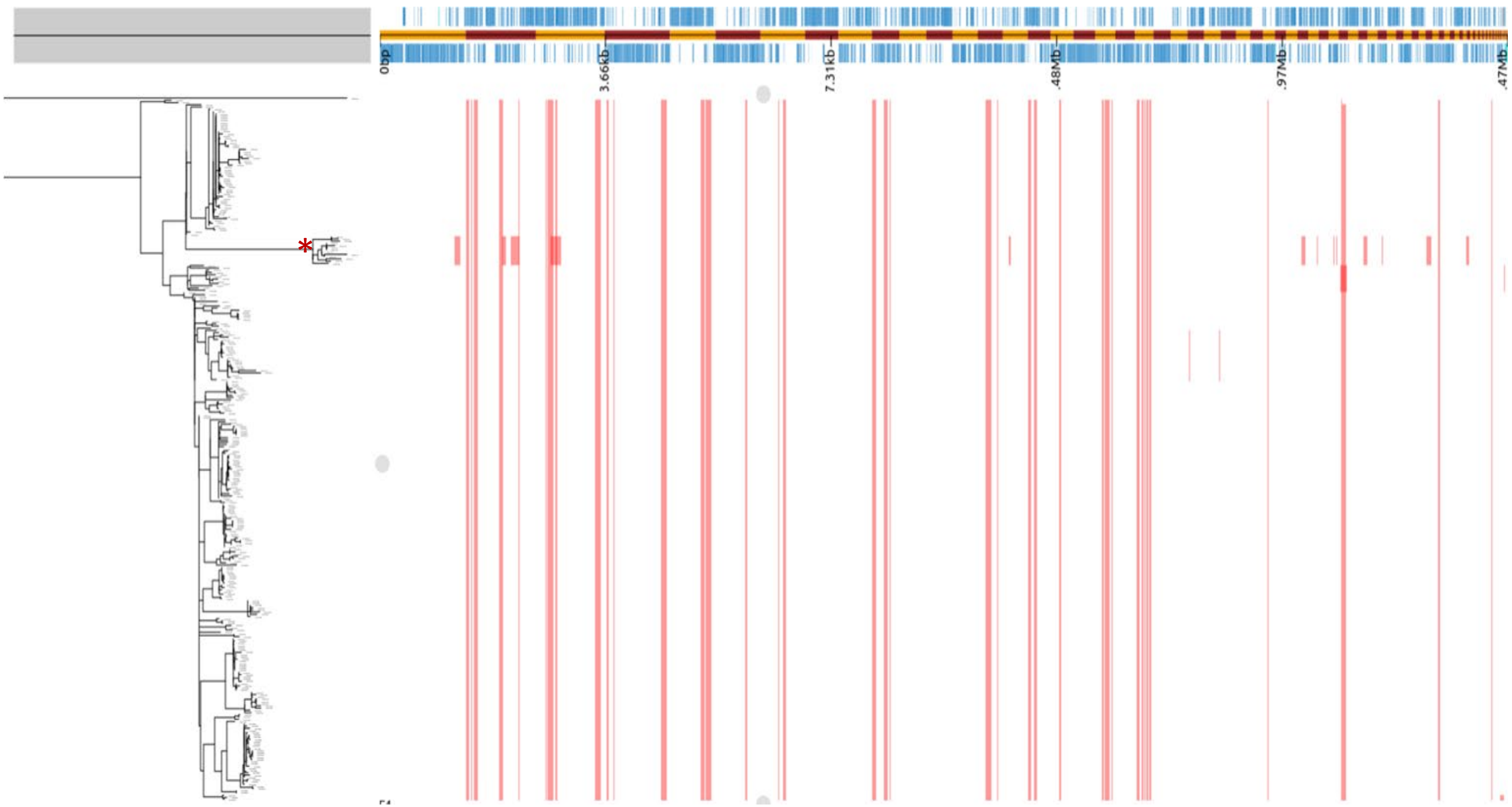

Figure S2. French ST25 clonal population rooted Maximum Likelihood phylogeny based on substitutions in the core genome obtained after removal of recombination events and recombination map. The recombinant clade is indicated by the “*”.

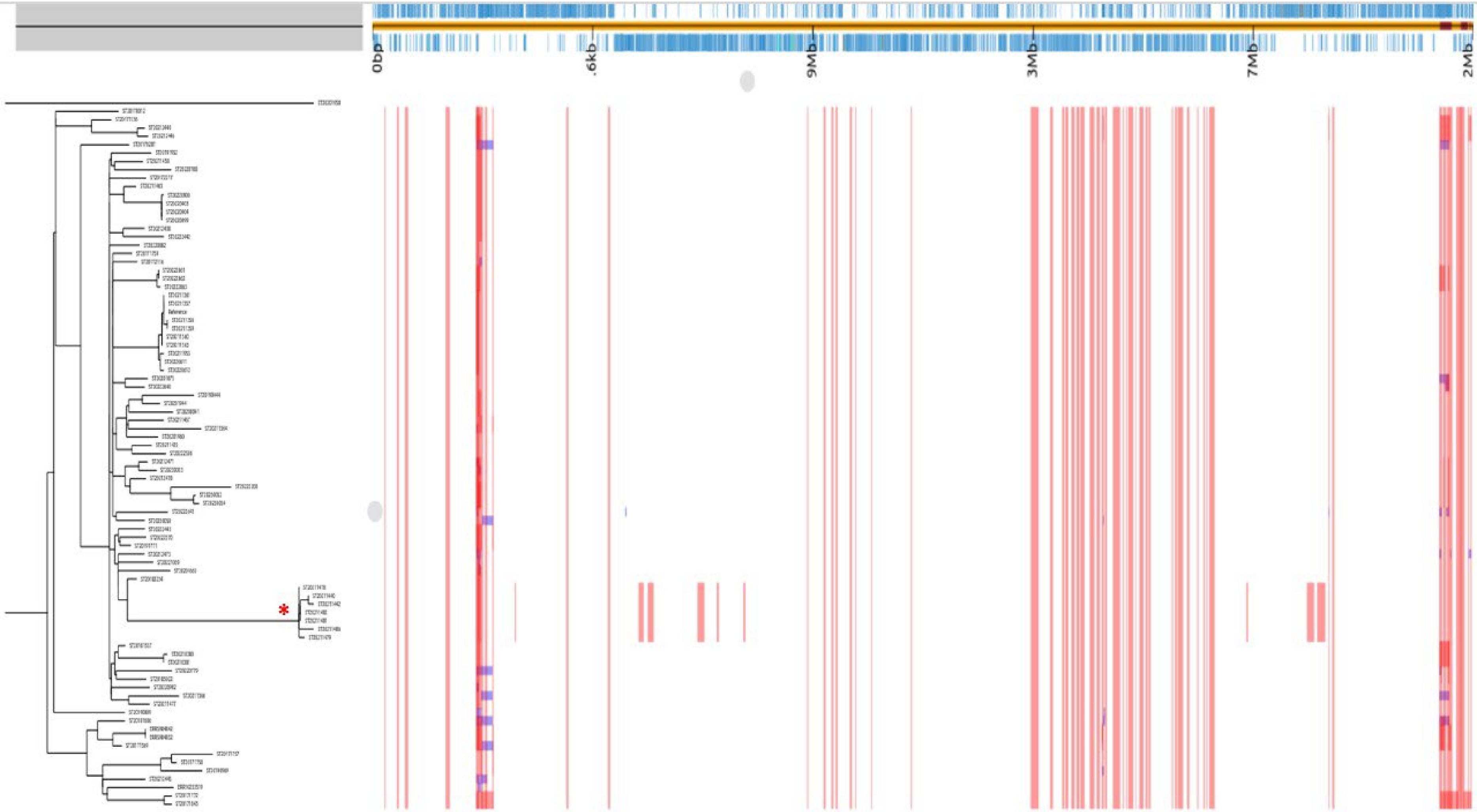

Classified as ECDC NORMAL
